# Mapping the evidence on exercise-related ventricular–arterial coupling adaptations in male adults: a scoping review

**DOI:** 10.3389/fcvm.2026.1807658

**Published:** 2026-05-20

**Authors:** Inês Noronha Cipriano, Hannes Holm, Peter M. Nilsson, Pedro Valente, Telmo Pereira, Luís Manuel Rama

**Affiliations:** 1 FCDEF, University of Coimbra, Coimbra, Portugal; 2 CIPER, University of Lisbon, Lisboa, Portugal; 3 Department of Clinical Sciences, Lund University, Malmö, Sweden, Malmö, Sweden; 4 Department of Cardiology, Skåne University Hospital, Malmö, Sweden; 5 CiBB - Center for Innovative Biomedicine and Biotechnology, University of Coimbra, Coimbra, Portugal; 6 CNC- Center for Neuroscience and Cell Biology, University of Coimbra, Coimbra, Portugal; 7 Polytechnic University of Coimbra, Coimbra, Portugal; 8 H&TRC - Health & Technology Research Center, Coimbra Health School, Polytechnic University of Coimbra, Coimbra, Portugal

**Keywords:** arterial stiffness, cardiac efficiency, exercise, physical activity, ventricular arterial coupling

## Abstract

**Background:**

Ventricular–arterial coupling (VAC) integrates cardiac and vascular function and has emerged as a relevant marker of cardiovascular efficiency and adaptability. Exercise and physical activity are believed to influence VAC through multiple physiological pathways, yet the scope, methods, and consistency of available evidence remain unclear.

**Objective:**

To map and characterize the existing evidence on the relationship between exercise, physical activity, and VAC across male adult populations, assessment methods, and study designs.

**Methods:**

A scoping review was conducted in accordance with JBI methodology and reported following PRISMA-ScR guidelines. Studies published between 1990 and 2025 were identified through PubMed, Scopus, Web of Science, and the Cochrane Library. Eligible studies examined VAC in male adults in relation to exercise or physical activity using invasive or non-invasive methods. Consistent with scoping review methodology, diverse study designs were included, and no formal risk-of-bias assessment was performed.

**Results:**

Eleven studies involving 2,229 participants were included. Evidence encompassed interventional, observational, and methodological studies conducted in male healthy adults, athletes, older individuals, and populations with cardiovascular risk. VAC was assessed using multiple approaches, most commonly the arterial elastance/end-systolic elastance (Ea/Ees) ratio and pulse wave velocity/global longitudinal strain (PWV/GLS). Exercise exposure ranged from acute stress testing to structured training and lifelong physical activity. Substantial heterogeneity was observed in study designs, exercise contexts, and VAC measurement techniques. Across studies, physically active and endurance-trained individuals demonstrated preserved or optimised VAC, with aerobic exercise associated with improved ventricular efficiency and reduced arterial load, while VAC remained relatively stable during acute haemodynamic stress.

**Conclusions:**

The literature relating exercise and VAC is conceptually and methodologically heterogeneous. Available evidence suggests that aerobic exercise and sustained physical activity are associated with preserved or favourable VAC profiles across male adult populations; however, causal inferences remain limited. This scoping review highlights important methodological gaps and provides a framework to guide future mechanistic and longitudinal research.

## Introduction

Cardiovascular disease remains the leading cause of morbidity and mortality worldwide, with physical inactivity recognised as a major modifiable risk factor (1). Regular physical activity and structured exercise are associated with favourable profiles in myocardial performance, vascular function, and overall haemodynamic efficiency (2, 3). An integrative index that captures the interaction between these cardiac and vascular components is ventricular–arterial coupling (VAC), which describes the relationship between left ventricular contractility and arterial load (4, 5).

VAC provides a systems-level view of cardiovascular performance, reflecting how efficiently the heart and arterial system interact to optimise stroke work and energetic efficiency (2, 6). VAC is commonly quantified through the ratio between arterial elastance (Ea) and end-systolic elastance (Ees). Ea represents the effective arterial load imposed on the left ventricle and is typically estimated as the ratio between end-systolic pressure and stroke volume, while Ees reflects ventricular contractile stiffness derived from pressure–volume relationships. The Ea/Ees ratio therefore provides an integrated representation of ventricular contractility and arterial load. Values close to unity are generally interpreted as reflecting efficient mechanical coupling between the ventricle and the arterial system, whereas deviations may indicate increased arterial load or altered ventricular contractile performance. In healthy individuals, Ea/Ees, is typically close to 1.0, reflecting an efficient matching between left ventricular contractility and arterial load (3). In addition to the classical Ea/Ees ratio, several surrogate indices have been used to characterise VAC non-invasive settings. Composite measures such as the PWV/GLS ratio integrate arterial stiffness and myocardial deformation, thereby providing an indirect assessment of ventricular–vascular interaction (7).

Increased PWV/GLS was associated with older age (2, 8, 9), and a higher degree of cardiovascular risk factors (i.e., BMI, blood pressure, LDL, diabetes, hypertension) (7), as well as with physical conditioning and athletic training (6). Environmental and physiological stressors may also influence ventricular–arterial interaction. For example, recent work has shown that environmental adaptations such as heat acclimation can modify cardiovascular and haemodynamic responses, highlighting how systemic stressors may affect ventricular–vascular dynamics beyond traditional cardiovascular disease contexts (10). Consequently, VAC has gained attention as a physiological marker capable of capturing both adaptive and maladaptive cardiovascular responses. Exercise may influence VAC through multiple mechanisms, including reductions in arterial stiffness, improvements in endothelial function, and modulation of ventricular elastance.

Alterations in VAC may occur in both acute and chronic contexts. Acute responses are commonly observed during exercise or haemodynamic stress, when ventricular contractility and arterial properties adjust dynamically to maintain stroke work and cardiovascular efficiency. In contrast, chronic changes may arise from long-term exposure to physical training, ageing, or cardiometabolic risk factors, leading to structural and functional modifications in both ventricular performance and arterial stiffness. Distinguishing between these contexts is important when interpreting exercise-related VAC findings, as acute physiological responses may differ from longer-term cardiovascular adaptations.

The existing literature is characterized by marked heterogeneity. Studies differ widely in exercise exposure (acute exercise, structured training, lifelong activity), populations studied (healthy individuals, athletes, older adults, clinical groups), and methods used to estimate VAC. Furthermore, some studies examine exercise as an intervention (2), while others assess VAC during exercise testing or in relation to habitual physical activity (11).

Given this diversity, a scoping review is the most appropriate approach to comprehensively map the available evidence, clarify key concepts, and identify methodological gaps. The present scoping review therefore aims to characterise how exercise and physical activity have been studied in relation to ventricular–arterial coupling across male adult populations and research contexts.

## Methods

### Study design

This scoping review was conducted in accordance with the Joanna Briggs Institute (JBI) methodology for scoping reviews (12) and is reported following the PRISMA-ScR guidelines (Preferred Reporting Items for Systematic Reviews and Meta-Analyses extension for Scoping Reviews) (13). In contrast to systematic reviews designed to evaluate intervention effectiveness, scoping reviews aim to map the breadth and characteristics of the available evidence. Therefore, the objective of the present review was not to determine causal effects of exercise on ventricular–arterial coupling (VAC), but rather to characterise how VAC has been investigated across different exercise contexts, populations, and methodological approaches.

### Review question

The review was guided by the Population–Concept–Context (PCC) framework:

- Population: Male adults (≥18 years), including healthy individuals, athletes, older adults, and populations with cardiovascular risk or disease.
- Concept: Ventricular–arterial coupling
- Context: Exercise, physical activity, exercise testing, or training interventions

The guiding question was: How has ventricular–arterial coupling been investigated in relation to exercise and physical activity in male adult populations?

### Eligibility criteria

Studies were eligible for inclusion if they:

- Included male adult participants (≥18 years);
- Examined ventricular–arterial coupling;
- Assessed VAC at rest, during exercise, following exercise, or in relation to structured training or habitual physical activity;
- Used invasive or non-invasive measurement techniques;
- Were original research articles published in English between 1990 and 2025.

Studies were excluded if they:

- Focused exclusively on pediatric populations;
- Did not examine VAC in any exercise- or activity-related context;
- Were narrative reviews, conference abstracts, or editorials.

Health status, and study design were not used as exclusion criteria, consistent with the exploratory nature of a scoping review.

### Search strategy

Electronic searches were conducted in PubMed, Scopus, Web of Science, and the Cochrane Library. Search terms combined controlled vocabulary and free-text terms related to ventricular–arterial coupling and exercise, including: “ventricular–arterial coupling”, “arterial elastance”, “end-systolic elastance”, “exercise”, “physical activity”, and “cardiovascular stress”.

### Study selection

**Figure 1:**
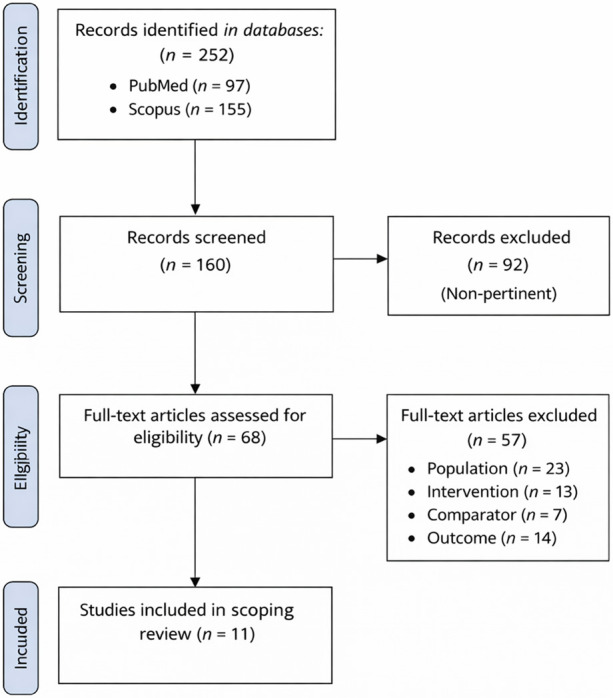
PRISMA flow diagram for scoping reviews (PRISMA-ScR).

Duplicates were removed using citation management software (Rayyan platform). Titles and abstracts were independently screened by two reviewers using the Rayyan platform (https://rayyan.ai/users/sign_in), a web-based tool designed for systematic review management. Conflicts in selection were resolved through discussion or consultation with a third reviewer. Articles passing the initial screening were screened for eligibility based on inclusion and exclusion criteria. The study selection process is summarized in the PRISMA (PRISMA-ScR) flow diagram (14) (Figure 1).

### Data charting and synthesis

Data were extracted using a predefined charting form, including study design, population characteristics, exercise context, VAC assessment method, and key findings. Consistent with JBI guidance (12), no formal risk-of-bias assessment was undertaken, as the objective was to map the evidence rather than to evaluate intervention effectiveness. Findings were synthesised descriptively and organised into thematic categories.

## Results

### Study characteristics

Eleven studies published between 1995 and 2025 were included, comprising a total of 2,229 participants (Table 1). Sample sizes ranged from 8 to 1,333 individuals. Study designs included interventional trials, cross-sectional observational studies, and methodological validation studies. Populations studied included healthy young adults, endurance-trained athletes, sedentary individuals, older adults, and cohorts with cardiovascular risk factors such as hypertension or coronary artery disease.

**Table 1:** Study characterization in terms of objective and findings.

Year	Study	Population	Objectives	Findings
1995	(15)	8 Healthy, 24 LV dysfunction patients	Validate non-invasive pressure-volume loop measurements for VAC assessment in healthy and LV dysfunction patients.	Non-invasive methods validated; healthy individuals maintained VAC during stress; LV dysfunction patients showed impairment.
2012	(14)	148 Adults (39.2% hypertensive)	Examine VAC impairment and myocardial work in hypertensive and normotensive during isometric stress.	Hypertension impaired VAC and cardiac reserve during stress; non-invasive methods validated.
2012	(13)	21 Sedentary obese adults	Compare the effects of aerobic and resistance training on VAC and vascular function in sedentary obese adults.	Aerobic training enhanced VAC, vascular function, and cardiac efficiency more than resistance training.
2013	(19)	23 Endurance-trained individuals	Investigate the effects of high-intensity and steady-state exercise on VAC and gender differences.	VAC remained stable post-exercise; linked to aerobic fitness;
2016	(16)	36 Coronary artery disease patients, 18 healthy controls	Evaluate carotid elasticity and left ventricular elastance during graded exercise in coronary artery disease patients and controls.	Healthy controls showed dynamic carotid and ventricular adaptation; coronary artery disease patients exhibited impaired VAC.
2018	(4)	12 Young sedentary, 12 elderly sedentary, 11 athletes	Evaluate the effects of endurance training on the dynamic Starling mechanism and VAC in different age groups.	Dynamic Starling mechanism gain improved by 65% post-training; age effects not entirely restored.
2018	(5)	102 Seniors categorized by exercise habits	Determine the effects of lifelong exercise training dose on VAC and age-related decline in seniors.	≥4–5 Sessions/week preserved VAC; reduced age-related decline in older adults.
2020	(2)	21 Older adults (mean age 81)	Assess the impact of a 3-month personalized exercise program on VAC and arterial stiffness in older adults.	Significant improvement in VAC, arterial stiffness, and cardiac efficiency in the intervention group.
2022	(6)	598 Elite athletes (348 males, 250 females)	Identify sport-specific cardiac adaptations using VAC metrics and clustering techniques in elite athletes.	VAC remained optimal; clustering revealed sport-specific cardiac adaptations.
2023	(7)	1,333 Middle-aged individuals	Examine associations between VAC parameters (Ea/Ees, PWV/GLS) and cardiovascular risk factors in a large cohort.	PWV/GLS was strongly associated with cardiovascular risk factors; Ea/Ees inversely correlated with age.
2023	(17)	13 Endurance-trained and 10 sedentary controls	Compare VAC efficiency between endurance-trained individuals and sedentary controls using pressure-volume loops.	Endurance training improved VAC and myocardial efficiency compared to sedentary controls.

Characteristics and key findings of the studies included in this scoping review. The table summarises the main characteristics of the included studies investigating ventricular–arterial coupling (VAC) in relation to exercise and physical activity in male adult populations. For each study, the year of publication, study population, primary objective, and principal findings are reported. The studies include interventional trials, observational analyses, and methodological investigations examining VAC responses during exercise, training interventions, or habitual physical activity.

### Exercise and physical activity contexts

Four main exercise-related contexts were identified:

1. Structured exercise interventions, including aerobic training, resistance training, and combined programs (2, 4, 13);
2. Acute exercise or stress testing, such as graded cycling or isometric handgrip (9, 14–16);
3. Habitual or lifelong physical activity, assessed through training history or activity levels (5, 6, 17);
4. Observational, Large cohorts without exercise manipulation (7).

### VAC assessment methods

VAC was assessed using diverse approaches (Table 2). The most common method was the non-invasive estimation of the Ea/Ees ratio, derived from echocardiographic and blood pressure measures (6, 7, 9, 13–15, 17). Other studies employed surrogate indices such as PWV/GLS ratios or assessed related constructs such as the dynamic Starling mechanism (4, 17, 18). Methodological variability was substantial, with differences in assumptions, modelling techniques, and physiological conditions under which VAC was assessed.

**Table 2:** Study characterization.

Year	Study	Study design	Exercise intervention	VAC measurement methods
1995	(15)	Intervention	Exercise stress test	Relative indices of Ees and Ea/Ees derived from non-invasive measurements
2012	(14)	Cross-sectional	Isometric handgrip exercise	Ea/Ees
2012	(13)	Intervention	6 Months of either Aerobic Training (AT) or Resistance Training (RT)	Ratio of ventricular-to-arterial elastance. Strain derived variables.
2013	(19)	Intervention	Low-intensity steady-state (SS) and high-intensity (HI) exercise bouts.	Ea/Ees. Ea calculated as ESP/stroke volume; Ees calculated as ESP/end-systolic volume
2016	(16)	Cross-sectional	Exercise stress echocardiography	LV elastance, carotid distensibility.
2018	(5)	Cross-sectional	Not applicable	Ea, cf-PWV (carotid-femoral pulse wave velocity) and dynamic Starling mechanism
2018	(4)	Cross-sectional and Longitudinal	Longitudinal: 3 months of exercise training.	Dynamic Starling mechanism was assessed.
2020	(8)	Non-randomised pilot study	3-month multidisciplinary intervention, with physical exercise component in Group 2	Ea. Pulse Wave Analysis (PWA) to assess central hemodynamic parameters.
2022	(6)	Cross-sectional	Various sports with different dynamic and static loads	Ea/Ees
2023	(7)	Cross-sectional comparative observational study	Graded semi-supine cycling ramp protocol	Non-invasive pressure-volume loop analysis of Ea and Ees (single-beat estimation method), PWV/GLS ratio
2023	(17)	Large cross-sectional population study	No exercise intervention (habitual activity observation)	Ea/Ees ratio via echocardiography, PWV/GLS ratio

Methodological characteristics of the studies included in the scoping review. This table summarises the methodological approaches used in the included studies, including study design, exercise exposure or intervention, and the techniques used to estimate ventricular–arterial coupling (VAC). Methods include non-invasive pressure–volume loop approximations, arterial elastance/end-systolic elastance (Ea/Ees) ratios, pulse wave analysis (PWA), pulse wave velocity (PWV), and indices derived from echocardiography and myocardial strain analysis. The table highlights the methodological diversity of VAC assessment across exercise physiology and cardiovascular research contexts.

### Mapping of key findings

Across studies, physically active individuals and endurance-trained athletes generally exhibited preserved or optimised VAC profiles. Structured aerobic exercise interventions were associated with reductions in arterial elastance and improved ventricular efficiency in some populations, particularly older adults (2). Acute exercise studies mapped that VAC is dynamically regulated during stress, often remaining stable despite increases in haemodynamic load (9, 15). Observational studies in larger cohorts highlighted associations between VAC indices and cardiovascular risk factors, underscoring the clinical relevance of ventricular–arterial interaction even in ostensibly healthy populations (7).

Across intervention studies, exercise training was generally associated with favourable ventricular–arterial coupling profiles, although the magnitude of change varied depending on exercise modality and participant characteristics. Aerobic training interventions in sedentary or older adults were associated with reductions in arterial elastance and improved indices of ventricular efficiency, suggesting that endurance-oriented exercise may enhance ventricular–vascular interaction through simultaneous improvements in arterial compliance and myocardial performance (13, 18). In contrast, resistance training interventions showed more heterogeneous responses, indicating that different exercise modalities may influence VAC through distinct physiological mechanisms (13).

Several studies examined VAC responses during acute haemodynamic stress, including graded cycling protocols and isometric handgrip exercise. These investigations suggested that VAC-related indices may remain relatively stable despite increases in cardiac output and arterial load (14–16). This stability reflects coordinated adjustments between ventricular contractility and arterial properties, allowing the cardiovascular system to maintain efficient coupling during periods of increased physiological demand (19).

Population characteristics also appear to influence ventricular–arterial interaction. Observational studies reported associations between VAC indices and cardiometabolic risk factors, including hypertension and ageing (7). In older adults, higher levels of habitual physical activity were associated with more favourable coupling profiles and reduced age-related deterioration in cardiovascular performance (3, 9). These findings highlight the potential role of VAC as an integrative marker linking physical activity, cardiovascular ageing, and cardiometabolic health.

Studies involving endurance-trained individuals and elite athletes consistently reported preserved or optimized VAC profiles despite higher cardiovascular workloads during exercise (6, 17). These findings suggest that long-term exposure to exercise training may promote adaptive cardiovascular remodeling that maintains efficient ventricular–vascular interaction under both resting and exercise conditions.

## Discussion

This scoping review mapped the existing literature on ventricular–arterial coupling in the context of physical exercise, revealing substantial heterogeneity in study designs, populations, and assessment methodologies. Regarding the breadth of evidence linking exercise, physical activity, and ventricular–arterial coupling in adults, rather than providing definitive conclusions on effectiveness, the findings illustrate how VAC has been investigated across diverse contexts and highlight the complexity of interpreting exercise-related adaptations.

From a physiological perspective, an optimized VAC state refers to a balance between ventricular contractility and arterial load that maximizes stroke work while minimizing myocardial energetic cost. In physically active individuals and athletes, favourable coupling profiles may arise from reduced arterial stiffness, improved endothelial function and preserved ventricular contractile reserve. Previous work has described supranormal cardiac function in endurance athletes associated with better arterial and endothelial function (20). Other studies examining endurance exercise have reported transient alterations in ventriculo-arterial coupling following prolonged exercise, reflecting the dynamic interaction between cardiovascular stress and adaptive reserve (21).

### Physiological pathways linking exercise and VAC

Across structured training interventions, aerobic exercise exposure was reported alongside more favourable VAC-related indices in older adults and in a sedentary obese cohort, while resistance training showed less consistent patterns (2, 13).

Acute exercise studies suggested that VAC-related indices may remain stable immediately following high-intensity or steady-state exercise in endurance-trained adults, supporting the idea that short-term responses may differ from longer-term training adaptations (19, 22).

In observational contexts, habitual activity and lifelong exercise dose were associated with preserved cardiovascular performance during exercise and with beneficial VAC-related profiles in older adults, although these designs cannot establish training effects (5, 9).

In elite athletes, VAC-based phenotyping and clustering approaches suggested sport-specific cardiac adaptation patterns while maintaining overall coupling profiles compatible with high-performance cardiovascular function (6).

Large cohort evidence further indicated that VAC-related parameters, including PWV/GLS, were associated with cardiovascular risk factor profiles in middle-aged adults, highlighting that VAC metrics may capture variation linked to cardiometabolic risk beyond exercise exposure alone (7).

### Methodological heterogeneity and conceptual challenges

The reviewed literature is characterised by substantial heterogeneity in VAC assessment methods, exercise exposure, and populations studied. Differences in measurement techniques and analytical frameworks limit direct comparisons and impede synthesis of causal effects. Moreover, many studies are observational or cross-sectional, restricting inference regarding training-induced adaptations.

Studies used different non-invasive approaches, including Ea/Ees derived from pressure-volume loop surrogates, coupling measures during stress testing, and combined indices integrating arterial stiffness and myocardial deformation such as PWV/GLS (7, 14–16).

Several studies assessed coupling under haemodynamic stress or during graded exercise, emphasising VAC as a dynamic property that may reveal physiological reserve or impairment under load rather than at rest (14–16).

The range of populations also contributes to conceptual complexity. The included studies encompassed healthy individuals, endurance-trained adults, elite athletes, older adults and coronary artery disease or hypertensive samples, which complicates interpretation of whether observed coupling profiles reflect adaptation, ageing, disease or their interaction (5, 14, 16, 19).

Collectively, this diversity in exposure definitions, measurement techniques and populations supports the need for clearer operational definitions and more standardised VAC assessment protocols in exercise physiology research.

### Implications for research and clinical physiology

The mapped evidence suggests that future studies should distinguish more explicitly between structured training interventions, acute exercise responses and habitual activity associations, as these contexts address different physiological questions and are not directly interchangeable (2, 5, 13, 19).

Longitudinal interventions designed with VAC as a primary outcome, paired with harmonised non-invasive assessment methods, would help clarify whether VAC changes represent true physiological adaptation or reflect measurement variability across protocols (2, 13, 15).

Given that PWV/GLS was strongly associated with cardiovascular risk profiles in a population cohort, future work could explore how composite coupling indices behave across exercise exposure gradients and risk strata, without assuming causal training effects (7).

## Limitations

This scoping review has several limitations. The inclusion of heterogeneous study designs and populations precludes quantitative synthesis. Sex-specific analyses were limited, reflecting gaps in the existing literature rather than selection bias. Additionally, the absence of formal quality appraisal aligns with scoping review methodology but limits evaluation of internal validity.

## Conclusions

By mapping the limited but methodologically diverse body of evidence available, this scoping review provides a structured overview of how ventricular–arterial coupling has been assessed in exercise-related research and identifies key gaps that warrant further investigation.

**Figure 2:**
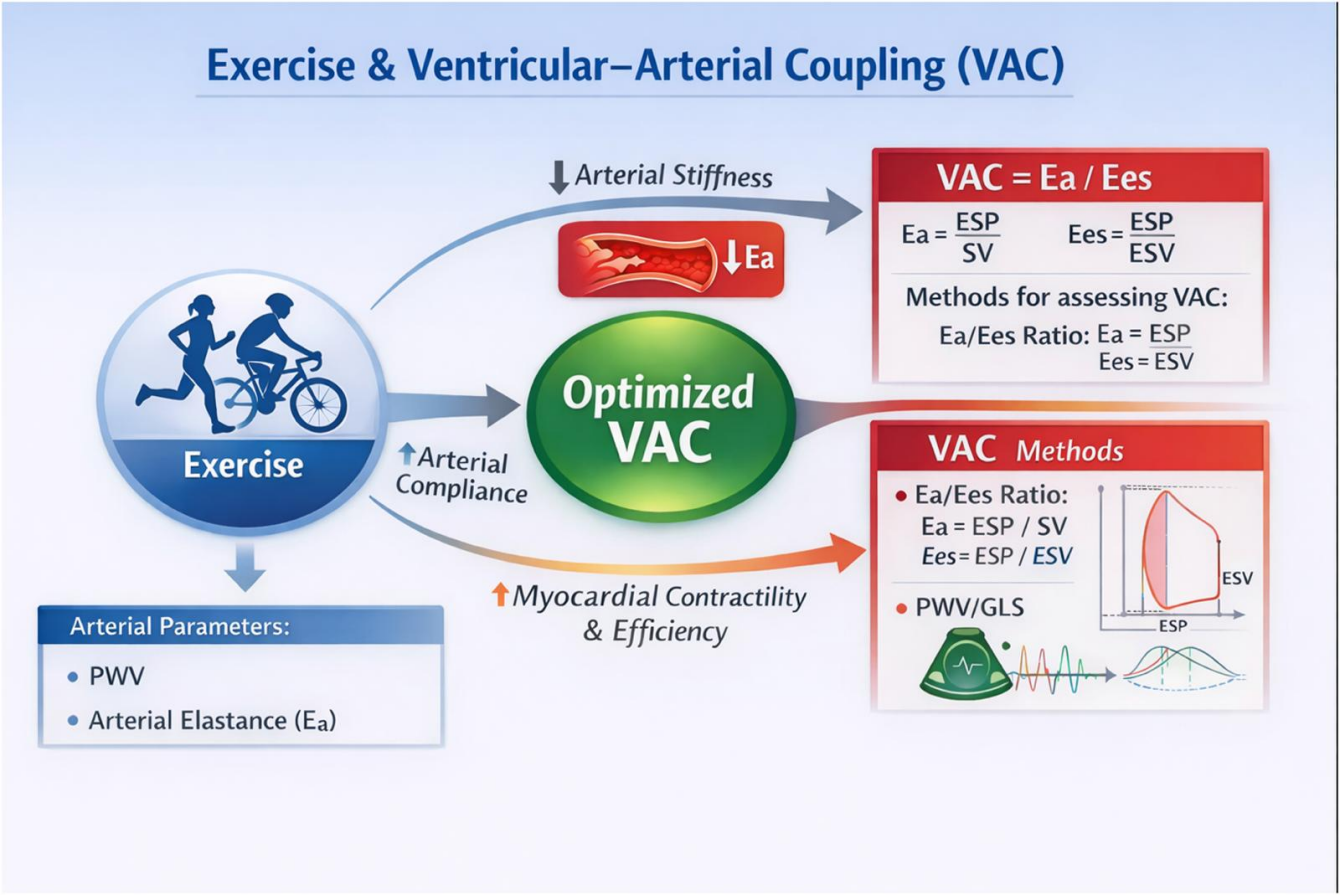
Conceptual overview of ventricular–arterial coupling (VAC) and its assessment in exercise physiology. Ventricular–arterial coupling reflects the interaction between left ventricular contractility and arterial load and represents an integrative marker of cardiovascular efficiency. VAC is commonly quantified using the ratio between arterial elastance (Ea) and end-systolic elastance (Ees). Ea represents the effective arterial load imposed on the ventricle, while Ees reflects ventricular contractile stiffness. Additional indices used in the literature include pulse wave velocity (PWV), stroke volume (SV), end-systolic pressure (ESP), end-systolic volume (ESV), and global longitudinal strain (GLS). These measures provide complementary information on arterial stiffness, myocardial deformation, and ventricular–vascular interaction under resting or exercise conditions.

This scoping review maps that the relationship between exercise, physical activity, and ventricular–arterial coupling has been investigated across a wide range of adult populations, exercise contexts, and methodological approaches. The available evidence suggests that aerobic exercise and sustained physical activity are associated with preserved or favourable VAC profiles, although causal mechanisms remain incompletely defined. Substantial methodological heterogeneity underscores the need for standardised assessment protocols and well-designed longitudinal studies to clarify the physiological and clinical significance of VAC in exercise science (Figure 2).
